# Silencing MicroRNA-155 Attenuates Cardiac Injury and Dysfunction in Viral Myocarditis via Promotion of M2 Phenotype Polarization of Macrophages

**DOI:** 10.1038/srep22613

**Published:** 2016-03-02

**Authors:** Yingying Zhang, Mengying Zhang, Xueqin Li, Zongsheng Tang, Xiangmin Wang, Min Zhong, Qifeng Suo, Yao Zhang, Kun Lv

**Affiliations:** 1 Laboratory Medicine of Yijishan Hospital, Wannan Medical College, Wuhu, 241001, PR China; 2 Central Laboratory of Yijishan Hospital, Wannan Medical College, Wuhu, 241001, People’s Republic of China; 3 Department of Pathology of Yijishan Hospital, Wannan Medical College, Wuhu, 241001, PR China; 4 Department of Biochemistry, Wannan Medical College, Wuhu, 241001, PR China

## Abstract

Macrophage infiltration is a hallmark feature of viral myocarditis. As studies have shown that microRNA-155 regulates the differentiation of macrophages, we aimed to investigate the role of microRNA-155 in VM. We report that silencing microRNA-155 protects mice from coxsackievirus B3 induced myocarditis. We found that microRNA-155 expression was upregulated and localized primarily in heart-infiltrating macrophages and CD4^+^ T lymphocytes during acute myocarditis. In contrast with wildtype (WT) mice, microRNA-155^−/−^ mice developed attenuated viral myocarditis, which was characterized by decreased cardiac inflammation and decreased intracardiac CD45^+^ leukocytes. Hearts of microRNA-155^−/−^ mice expressed decreased levels of the IFN-γ and increased levels of the cytokines IL-4 and IL-13. Although total CD4^+^ and regulatory T cells were unchanged in miR-155^−/−^ spleen proportionally, the activation of T cells and CD4^+^ T cell proliferation in miR-155^−/−^ mice were significantly decreased. Beyond the acute phase, microRNA-15^5−/−^ mice had reduced mortality and improved cardiac function during 5 weeks of follow-up. Moreover, silencing microRNA-155 led to increased levels of alternatively-activated macrophages (M2) and decreased levels of classically-activated macrophages (M1) in the heart. Combined, our studies suggest that microRNA-155 confers susceptibility to viral myocarditis by affecting macrophage polarization, and thus may be a potential therapeutic target for viral myocarditis.

Viral myocarditis (VM), an important cause of sudden cardiac death and heart failure in otherwise healthy young persons, is characterized by inflammation of the cardiac muscle. Similar to the human disease, infection of mice with *coxsackievirus B3* (CVB3) results in the development of acute myocarditis from days 7 to 14 post-infection^1^, which proceeds to autoimmune myocarditis and dilated cardiomyopathy (DCM) by day 35 post-infection^2^,^3^.

Although CVB3-induced myocarditis had been considered to be CD4^+^ T lymphocyte–mediated inflammatory heart disease^4^,^5^, accumulating data indicates that macrophages represent the major inflammatory infiltrates and play a pathogenic role in the development of VM. Macrophages, as master regulators of inflammation, are highly plastic and can differentiate into M1 (classically-activated) or M2 (alternatively-activated) macrophages^6^,^7^ depending on signaling conditions. M1 macrophages, induced by lipopolysaccharide (LPS) and interferon-γ (IFN-γ), typically produce copious amounts of pro-inflammatory cytokines (tumor necrosis factor [TNF]-α, interleukin [IL]-12) and generate reactive oxygen species (ROS). As such, M1 macrophages are associated with inflammation and tissue destruction. On the other hand, M2 macrophages, induced by Th2-produced IL-4 and IL-13, secrete high levels of anti-inflammatory cytokines (IL-10) and are characterized by increased arginase1 (Arg-1) activity and surface expression of macrophage mannose receptor (MMR, CD206) and macrophage galactosetype C-type lectin (MGL, CD301). Functionally, M2 macrophages show an anti-inflammatory phenotype associated with tissue repair and angiogenesis^8^,^9^,^10^,^11^,^12^,^13^.

Previous studies reported that the severity of myocardial inflammation correlates with the intensity of the macrophage infiltration^14^ and macrophage-depleted mice fail to develop VM^15^. However, substantial macrophage infiltration is not always indicative of severe myocarditis. Frisancho-Kiss *et al.*^1^ and Huber *et al.*^5^ observed an obvious macrophage infiltrate after CVB3 infection in female BALB/c mice which are less susceptible to VM. Furthermore, Li *et al.*^16^ found that adoptive transfer of *ex vivo*-programmed M1 macrophages, as expected exhibited significantly increased myocarditis, whereas transfer of M2 macrophages into susceptible male mice remarkably alleviated myocardial inflammation. Thus, it can be speculated that regulators of macrophage polarization may also exert pivotal functions in modulating heart inflammatory responses. However, despite the importance of this process to VM, the mechanisms underlying macrophage polarization remain to be explored.

MicroRNAs (miRNAs or miRs) are a class of short noncoding RNAs (18–25 nucleotides) that modulate gene expression at the post-transcriptional level and are involved in regulating several aspects of inflammation^17^,^18^. MiR-155 was among the first miRNAs linked to inflammation by virtue of its potent upregulation in multiple immune cell lineages by Toll-like receptor (TLR) ligands, inflammatory cytokines, and specific antigens^19^,^20^,^21^,^22^. A wide variety of immunologically relevant targets of miR-155 have been reported, implying distinct roles in mammalian immunity. The fitness of T regulatory cells is influenced by direct repression of suppressor of cytokine signaling 1 (SOCS1) by miR-155^21^. Overexpression of miR-155 in myeloid cells leads to myeloproliferative disorder through suppression of SHIP1 expression^22^. Besides, our previous study^23^ found that M1 macrophages demonstrated greater expression of miR-155 than M2 macrophages, suggesting functional importance for this miRNA in macrophage polarization. Despite these reported functions of miR-155 in both innate and adaptive immune cells, there has been little genetic evidence that endogenously expressed miR-155 indeed impacts inflammatory responses *in vivo* to date. Given the role of miR-155 in the differentiation of macrophages and the plasticity of macrophages in the development of VM, we aimed to examine whether miR-155 may affect cardiac inflammation and impact subsequent cardiac injury by regulating the inflammatory processes such as macrophage polarization. The present study provides genetic evidence to support a novel role of endogenous miR-155 in modulating macrophage polarization in a pattern that protects mice from CVB3-induced VM.

## Results

### miR-155^−/−^ mice develop attenuated CVB3-induced VM

**Figure 1:**
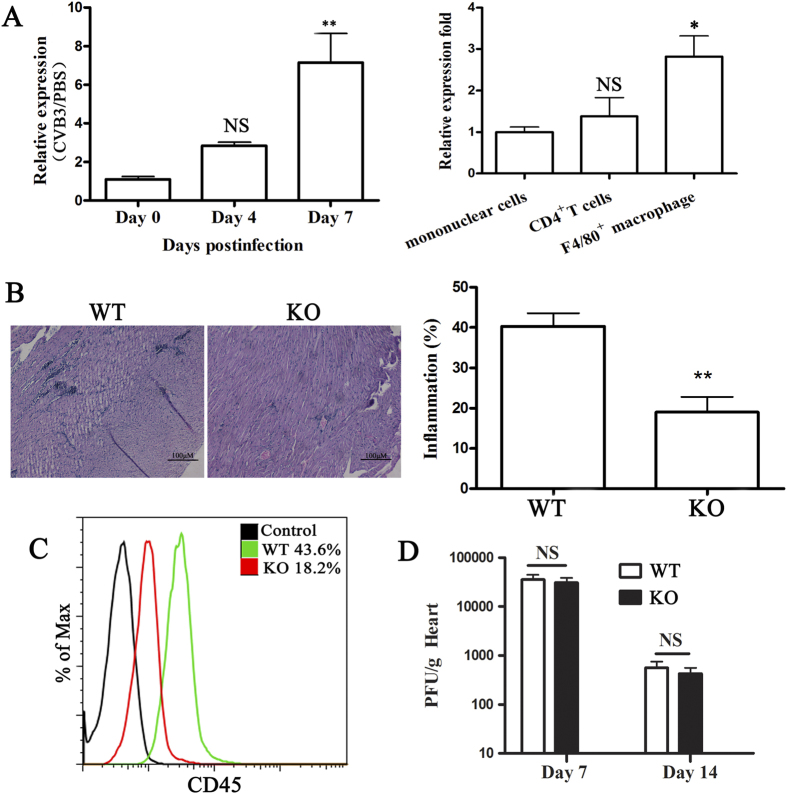
miR-155^−/−^ mice develop attenuated CVB3-induced VM. miR-155^−/−^ and WT mice received 1 × 10^5^ PFU of CVB3 or PBS i.p. on day 0 and the heart samples were isolated on day 7. (**A**) miR-155 expression was determined by real-time PCR in hearts or heart infiltrating cells from CVB3 or PBS injected WT mice. (**B**) Heart sections of CVB3-infected WT and miR-155^−/−^ KO mice were stained with H&E, and the severity of myocarditis was assessed as the percentage of the heart section with inflammation compared with the overall size of the heart section, with the aid of a microscope eyepiece grid. Scale bar = 100 μm. (**C**) Myocardial infiltration of CD45^+^ cells was determined by FACS. (**D**) Viral titers were measured using plaque assay. Experiments were repeated three times in triplicate with 8 mice per group. Bar graph data are presented as mean ± SD; *P < 0.05 and **P < 0.01 as compared with WT. WT: wildtype mice; KO: knockout mice; NS: no significance. Similar data presentation will appear in the subsequent figures.

To study the role of miR-155 in VM development, we first sought to analyze the expression of miR-155 in CVB3-infected wildtype (WT) mice during the course of VM. We found that miR-155 expression increased in heart tissue from CVB3 infected mice compared with PBS injected mice (Fig. 1A). Because CD4^+^ T cells and macrophages are the key mediators of pathogenesis in VM, we next analyzed the expression of miR-155 in heart-infiltrating mononuclear cells. We found that miR-155 expression was higher in CD4^+^ T cells and macrophages compared with total mononuclear cells (Fig. 1A). The potential role of miR-155 in VM was then examined using miR-155^−/−^ (KO) mice. Compared with WT mice, miR-155^−/−^ mice displayed a significant remission of VM showing few restricted mononuclear inflammation foci (Fig. 1B). To support this observation, lower frequency of CD45^+^ immune cells in the heart tissue of miR-155^−/−^ mice was demonstrated than in WT mice (Fig. 1C). Besides, we found no significant differences in viral replication in the hearts of miR-155^−/−^ and WT mice on day 7 after CVB3 infection (Fig. 1D).

### miR-155^−/−^ mice show improved mortality and cardiac functions during CVB3-induced VM

**Figure 2:**
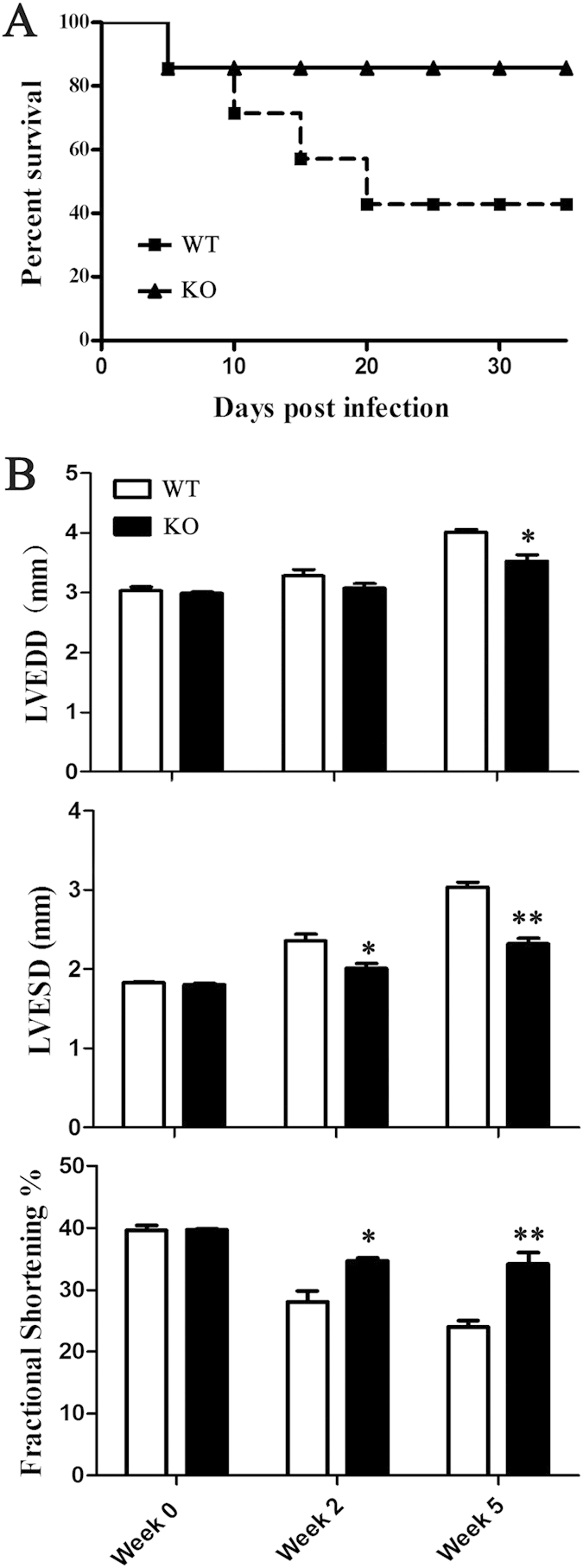
miR-155^−/−^ mice show improved mortality and cardiac function during CVB3-induced VM. miR-155^−/−^ and WT mice received 1 × 10^5^ PFU of CVB3 i.p. on day 0. (**A**) The survival rate of mice was observed until week 5 (day 35) post-infection. (**B**) Echocardiographic examination was performed at week 2 (day 14) and week 5 (day 35). Left ventricular (LV) diameters at end-diastole (LVEDD) and end-systole (LVESD) were measured, and fractional shortening (FS) was calculated. Experiments were repeated three times in triplicate with 15 mice per group. Bar graph data are presented as mean ± SD; *P < 0.05 and **P < 0.01 as compared with WT.

Silencing miR-155 could affect mortality, cardiac inflammation, and function in VM, miR-155^−/−^ and WT littermates were infected intraperitoneally with CVB3, and examined after 14 and 35 days. The absence of miR-155 resulted in decreased mortality, with approximately 85% mice surviving within 35 days of CVB3 infection, in comparison to 55% survival of the WT mice (Fig. 2A). In addition to decreased mortality, silencing miR-155 improved cardiac function in mice surviving after 5 weeks following CVB3 infection (Fig. 2B). miR-155^−/−^ mice that survived to this time point exhibited decreased left ventricular end diastolic dimension (LVEDD), decreased left ventricular end systolic dimension (LVESD), and increased fractional shortening (FS%). In conclusion, silencing miR-155 improved mortality and cardiac function during VM.

### miR-155^−/−^ mice express increased levels of anti-inflammatory cytokines during CVB3-induced VM

**Figure 3:**
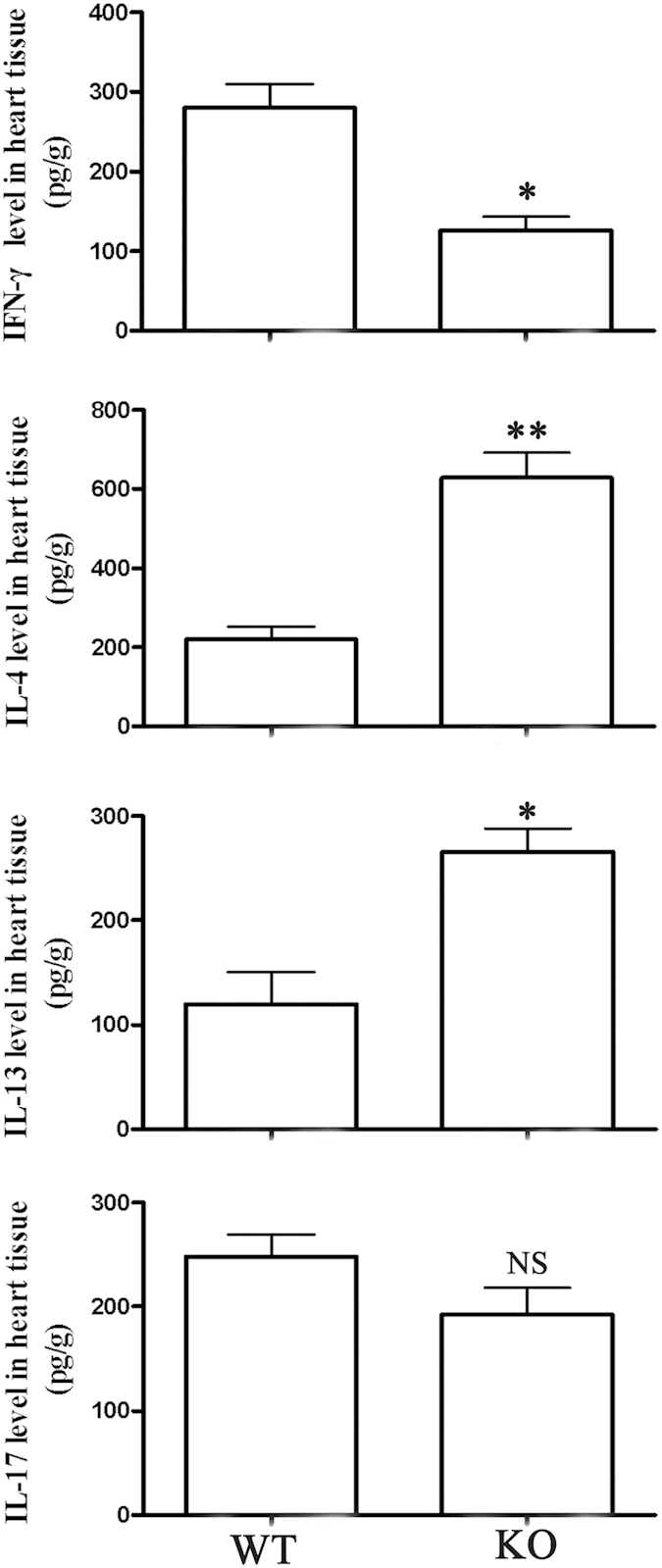
miR-155^−/−^ mice have increased levels of anti-inflammatory cytokines during CVB3-induced VM. miR-155^−/−^ and WT mice received 1 × 10^5^ PFU of CVB3 i.p. on day 0, and protein levels of IFN-γ, IL-4, IL-13, and IL-17 in the homogenized heart tissue were determined by ELISA on day 7 post-infection. Experiments were repeated three times in triplicate with 8 mice per group. Bar graph data are presented as mean ± SD; *P < 0.05 and **P < 0.01 as compared with WT.

To analyze changes in cytokine expression at day 7 of VM, we homogenized hearts of miR-155^−/−^ and WT mice and analyzed levels of the inflammatory cytokines including IFN-γ, IL-4, IL-13, and IL-17 by ELISA. CVB3 infection resulted in production of IFN-γ in WT mice, while silencing miR-155 led to significantly decreased IFN-γ level in myocardial tissue, but increased IL-4 and IL-13 level, indicating significant increased levels of anti-inflammatory cytokines (Fig. 3). Furthermore, we measured levels of intracardiac IL-17 to examine whether Th17 involved CD4^+^ T subsets in mediating cardiac pathology of CVB3-induced VM. However, we found that expression of IL-17 was not significantly altered in heart homogenates of miR-155^−/−^ and WT mice (Fig. 3). Thus, silencing miR-155 probably protects against VM by down-regulating pro-inflammatory cytokines and increasing levels of anti-inflammatory cytokines.

### miR-155^−/−^ mice display decreased spleen T cell activation during CVB3-induced VM

**Figure 4:**
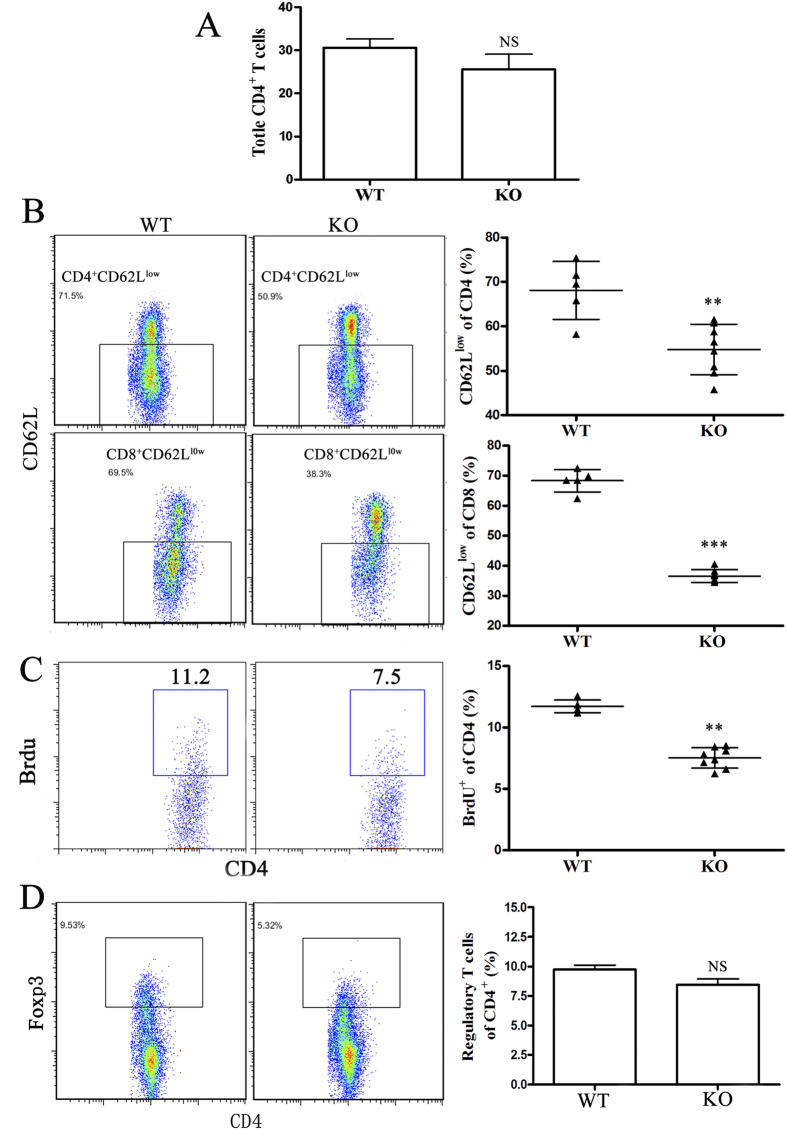
miR-155^−/−^ mice display decreased spleen T cell activation during CVB3-induced VM. miR-155^−/−^ and WT mice received 1 × 10^5^ PFU of CVB3 i.p. on day 0, and splenocytes were isolated on day 7. (**A**) The proportion of total CD4^+^ T cells was analyzed by FACS. (**B**) Activated CD4^+^ and CD8^+^ T cells were determined by surface CD62L^low^ expression. (**C**) 5-bromo-2-deoxyuridine (BrdU) was administered (0.8mg in 1ml PBS) 1 day before termination of mice. The proliferation of CD4^+^ T cells was determined by BrdU incorporation assay *in vivo*. (**D**) The proportion of CD4^+^CD25^+^Foxp3^+^ Tregs was analyzed by FACS. Experiments were repeated three times in triplicate with 10 mice per group. Bar graph data are presented as mean ± SD; **P < 0.01 and ***P < 0.001 as compared with WT.

The severity of myocarditis is largely influenced by the bias of CD4^+^ Th immune response. Therefore, we examined CD4^+^ T cell populations in miR-155^−/−^ splenocytes with VM. No differences were observed in the relative proportions of CD4^+^ T cells in the spleens of miR-155^−/−^ mice when compared to WT mice at day 7 (Fig. 4A). The effect of silencing miR-155 on the activation phenotype of T cells *in vivo* was also determined. Flow cytometric analysis of splenic T cells at day 7 demonstrated decreased T cell activation in miR-155^−/−^ mice, as determined by decreased proportions of CD4^+^ and CD8^+^ T cells expressing low levels of CD62L compared to WT mice (Fig. 4B). Furthermore, CD4^+^ T cells from miR-155^−/−^ and WT mice with CVB3-induced VM were assessed for proliferation by BrdU incorporation assay. As shown in Fig. 4C, CD4^+^BrdU^+^ effector T cells from miR-155^−/−^ mice 7 days post-infection significantly reduced compared to WT mice. To determine whether the attenuated VM in the absence of miR-155 was attributable to changes in Treg populations, we examined the proportions of CD4^+^CD25^+^Foxp3^+^ Tregs in the spleens of miR-155^−/−^ and WT mice on day 7 of CVB3-induced VM. We did not observe any differences in the proportion of Tregs in miR-155^−/−^ mice at this time-point (Fig. 4D). Thus, splenic T cells from miR-155^−/−^ mice were less activated and had lower proliferative responses than WT mice.

### Myocardial infiltrating macrophages exhibited a predominant M2 phenotype in miR-155^−/−^ mice during CVB3-induced VM

**Figure 5:**
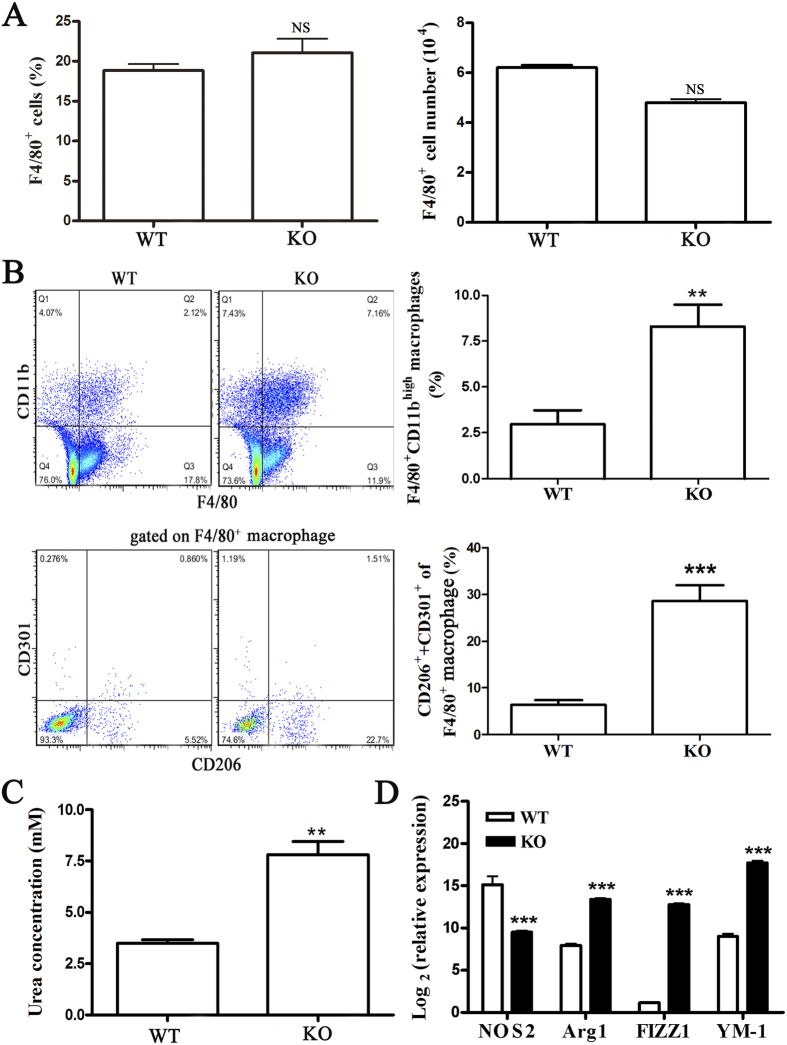
Phenotypic analysis of myocardial-infiltrating macrophages in miR-155^−/−^ and WT mice during CVB3-induced VM. miR-155^−/−^ and WT mice received 1 × 10^5^ PFU of CVB3 i.p. on day 0, and hearts were collected on day 7 post-infection. Myocardial infiltrating leukocytes were isolated from the hearts after enzymatic digestion. (**A**) The percentage and absolute numbers of F4/80^+^ cells were analyzed by FACS. (**B**) Infiltrating CD45^+^ leukocytes are subsetted by bivariate analysis of F4/80 and CD11b expression, revealing distinct populations: UL, F4/80^−^CD11b^high^ monocytes; UR, F4/80^+^CD11b^high^ macrophages; CD206 and CD301 expression were then determined by FACS, based on gates set from F4/80^+^ macrophages. (**C**) Arginase activity of sorted F4/80^+^ macrophages was assessed by an assay of urea production from arginine substrate and was normalized to cell counts. (**D**) NOS2, Arg1, FIZZ1, and YM-1 of sorted F4/80^+^ macrophages were determined by real-time PCR. Experiments were repeated three times in triplicate with 10 mice per group. Bar graph data are presented as mean ± SD; **P < 0.01 and ***P < 0.001 as compared with WT.

Accumulating data has indicated that abundant macrophages are recruited into the myocardium of male mice after CVB3 infection, which plays an indispensable role in the development of VM^14^,^15^,^24^. Therefore we next determined the frequency and the absolute numbers of macrophages accumulating in the myocardium using cell yield analysis of F4/80^+^ cells. We found that there were no differences in the proportions and the absolute numbers of F4/80^+^ macrophages in the hearts of miR-155^−/−^ and WT mice (Fig. 5A). Surprisingly, F4/80^+^CD11b^high^ macrophages were proportionally increased in miR-155^−/−^ hearts (Fig. 5B). Given that our data had thus far suggested that miR-155^−/−^ mice were less prone to VM, we speculated that functionally distinct macrophages may exist in miR-155^−/−^ and WT mice. To explain whether this relative increase in F4/80^+^CD11b^high^ macrophages corresponded to the decrease of pro-inflammatory cytokines in miR-155^−/−^ hearts, we further examined the phenotype of macrophages in miR-155^−/−^ mice heart infiltrates. CD206 and CD301 are markers of M2 macrophages. We observed increased proportions of F4/80^+^ macrophages bearing CD206 and/or CD301 in the hearts of miR-155^−/−^ mice at day 7 of CVB3-induced VM (Fig. 5B). Moreover, we observed strikingly decreased proportions of macrophages lacking both CD206 and CD301, which may indicate that classically-activated macrophages (M1) were decreased in hearts of miR-155^−/−^ mice (Fig. 5B). Furthermore, higher arginase activity, a typical enzymatic characteristic of M2 macrophages, was observed in F4/80^+^ macrophages from miR-155^−/−^ mice than in WT mice (Fig. 5C). Besides, F4/80^+^ macrophages derived from miR-155^−/−^ mice showed increased expression of M2-specific genes Arg1, FIZZ1, and YM1 compared with the macrophages isolated from WT mice (Fig. 5D). Taken together, these data indicated that miR-155 had substantial influence over the polarized differentiation of macrophage population in the heart during CVB3-induced VM.

### miR-155 plays a role in the development of macrophage polarization *in vitro*

**Figure 6:**
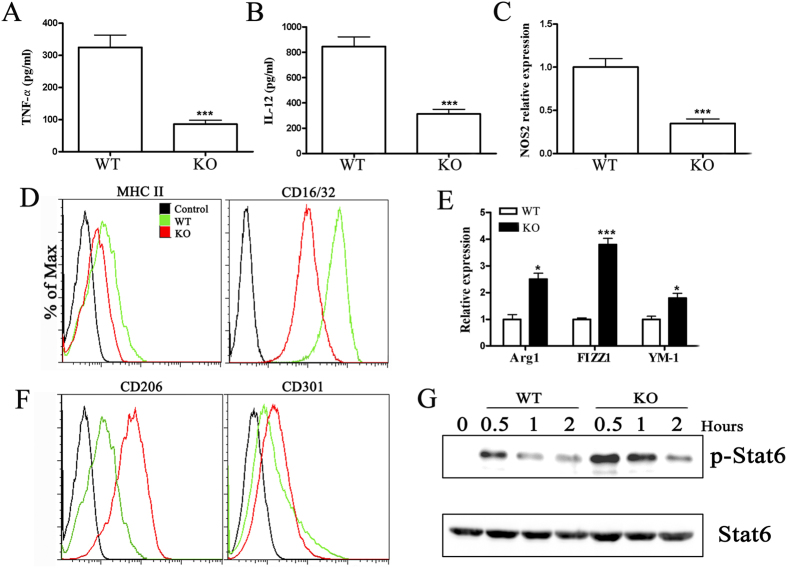
Silencing miR-155 promotes macrophage polarization toward M2 phenotype *in vitro*. Bone marrow cells were obtained by flushing the femurs from miR-155^−/−^ and WT mice, and bone marrow–derived macrophages (BMDMs) were prepared. Macrophage polarization was obtained by removing the culture medium and culturing cells for an additional 48h in RPMI 1640 supplemented with 5% FCS and 100 ng/ml LPS plus 20 ng/ml IFN-γ (for M1 polarization) or 20 ng/ml IL-4 (for M2 polarization). Then M1 and M2 macrophage–associated markers were analyzed, respectively (**A–D**) for M1, (**E–G**) for M2). (**A**) TNF-α and (**B**) IL-12 in the supernatant were assayed by ELISA. (C) The levels of NOS2 were determined by real-time PCR. (**D**) The surface levels of MHCII and CD16/32 were determined by FACS. (**E**) Arg1, FIZZ1, and YM-1 were determined by real-time PCR. (**F**) The surface levels of CD206 and CD301 were determined by FACS. (**G**) BMDMs from miR-155^−/−^ and WT mice were stimulated with IL-4 and collected after 30 min, 1 hour and 2 hour and subjected to Western blotting. Cells lysates were used to determine total STAT6 and p-STAT6 levels normalized against β-actin. Experiments were repeated three times in triplicate with 8 mice per group. Bar graph data are presented as mean ± SD; *P < 0.05 and ***P < 0.001 as compared with WT.

Macrophage polarization must need to properly receive and coordinate the signals provided by specific inflammatory cytokines that mediate their development. To determine whether miR-155 participates in macrophage polarization during CVB3-induced VM, bone marrow cells were obtained from miR-155^−/−^ and WT mice, and bone marrow–derived macrophages (BMDMs) were prepared. Macrophage polarization was achieved by culturing cells under M1 or M2 polarization conditions. As shown in Fig. 6A–C, the increases in TNF-α, IL-12, and NOS2 expression normally found after LPS treatment (M1-skewing condition) were diminished in miR-155^−/−^ BMDMs. In addition, miR-155 deficiency attenuated LPS-enhanced MHC II and CD16/32 levels, the markers of M1 macrophages, on the surface of miR-155^−/−^ BMDMs (Fig. 6D). These data suggested that miR-155 was a positive regulator of pro-inflammatory responses induced by TLR4 stimulation. Because IL-4 is a classical Th2 cytokine that induces M2 macrophage polarization^25^, we next evaluated the effect of miR-155 on IL-4–induced M2 polarization. We found that IL-4-induced expression of Arg1, FIZZ1, and YM-1 in miR-155^−/−^ BMDMs was significantly greater than in BMDMs of WT mice (Fig. 6E). Consistently, flow cytometric analysis revealed significantly increased CD206 and CD301 expression in miR-155^−/−^ BMDMs, which are markers of M2 phenotype (Fig. 6F). IL-4 stimulation induces STAT6 phosphorylation and translocation to the nucleus^26^,^27^, which is required for IL-4–induced M2 polarization. To determine whether silencing miR-155 enhanced STAT6 activation by IL-4, we also examined STAT6 phosphorylation in IL-4–treated miR-155^−/−^ and WT BMDMs. As expected, IL-4-induced STAT6 phosphorylation reached a peak after 30 min and decreased over the course of 2h in miR-155^+/+^ WT BMDMs, while silencing miR-155 resulted in increased levels of STAT6 phosphorylation (Fig. 6G). STAT6 total protein levels were not affected by silencing miR-155 (Fig. 6G). These data established that silencing miR-155 had a suppressive role in M1 macrophage polarization and drove macrophage polarization toward the M2 phenotype by enhancing the phosphorylation of STAT6.

### miR-155 antagonist treatment reduces the severity of CVB3-induced VM

**Figure 7:**
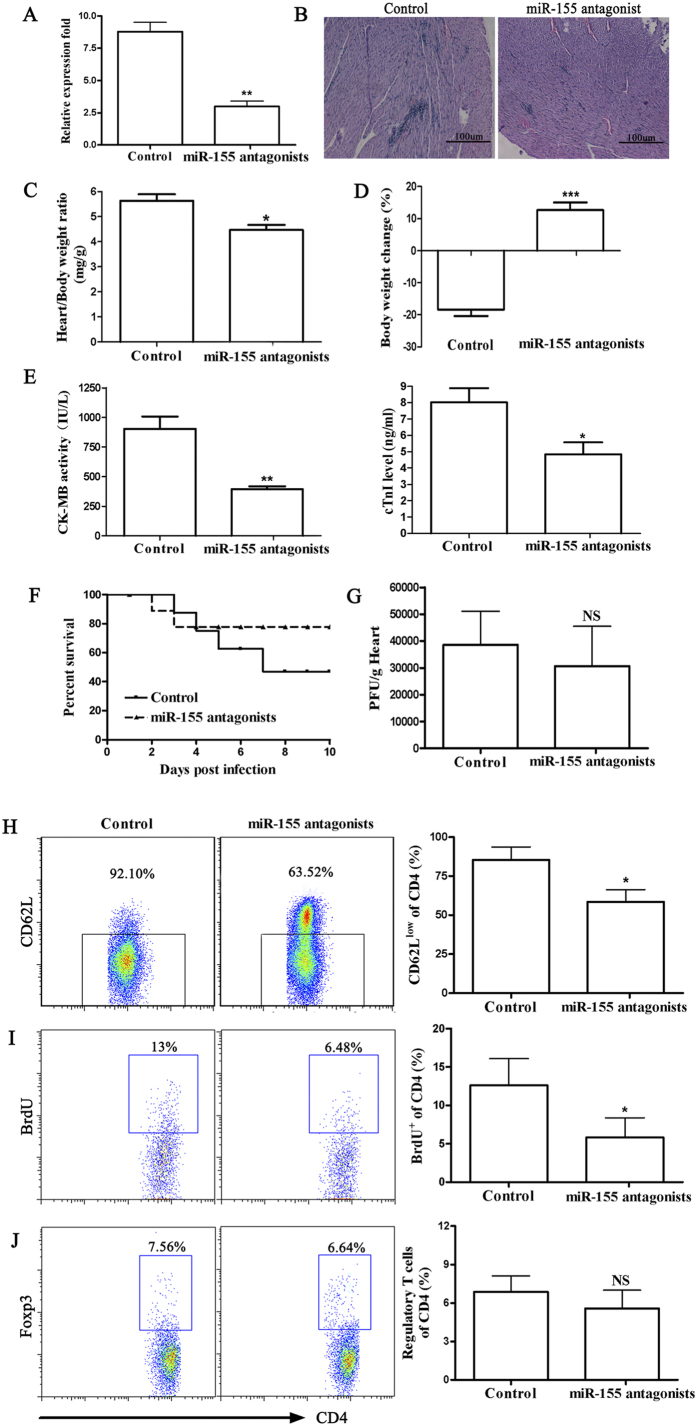
miR-155 antagonist reduces myocardial inflammation during CVB3-induced VM. C57BL/6 miR-155^+/+^ WT male mice received 1 × 10^5^ PFU of CVB3 i.p. on day 0. For *in vivo* anti–miR-155 treatment, 30 μl lipofectamine 2000 was mixed with miR-155 antagonist or scrambled control (25μg/mouse) dissolved in 170 μl PBS, and the liposome complexes were administered i.v. to mice 3 days prior to CVB3 infection. (**A**) miR-155 expression in hearts was determined by real-time PCR. (**B**) Heart sections were stained with H&E on day 7 (Scale bar = 100 μm). The parameters of the viral myocarditis were evaluated including heart weight (**C**), loss of body weight (**D**), activity of CK-MB and levels of cTnI (**E**) on day 7 post-infection. (**F**) The survival rate of mice was observed until day 10 post-infection. (**G**) Viral titers were measured using plaque assay. (**H**) Myocardial infiltrating leukocytes were isolated from the hearts after enzymatic digestion. Activated CD4^+^ T cells were determined by surface CD62L^low^ expression. (**I**) 5-bromo-2-deoxyuridine (BrdU) was administered (0.8mg in 1ml PBS) 1 day before termination of mice. The proliferation of CD4^+^ T cells was determined by BrdU incorporation assay *in vivo*. (**J**) The proportion of CD4^+^CD25^+^Foxp3^+^ Tregs was analyzed by FACS. Experiments were repeated three times in triplicate with 10 mice per group. Bar graph data are presented as mean ± SD; *P < 0.05, **P < 0.01 and ***P < 0.001 as compared with scrambled control.

Next, we investigated whether systemic administration of miR-155 antagonist *in vivo* three days prior to CVB3 infection affected the course of VM in miR-155^+/+^ WT mice. Therefore, we inhibited miR-155 *in vivo* during the first 7 days of VM. Three individual doses of miR-155 antagonist were administered i.v. to mice 3 days prior to CVB3 infection. The real-time PCR for miR-155 confirmed significant knockdown in the inflamed heart of miR-155 antagonist (25μg/mouse) treated VM mice (Fig. 7A).

At day 7 after infection, miR-155 inhibition resulted in alleviation of myocardial inflammation, reflected by restricted inflammation foci and decreased inflamed area (Fig. 7B). In agreement, miR-155 inhibition significantly attenuated heart weight increase and reduced body weight loss associated with systemic illness, as well as CK-MB activities and cTnI levels of serum (Fig. 7C–E). Furthermore, miR-155 inhibition significantly improved the survival rate from ~50% to 80% after CVB3 infection (Fig. 7F). In addition, we found no significant differences in viral replication in the hearts of VM mice on day 7 after miR-155 antagonist administration (Fig. 7G).

To better characterize the effects of miR-155 inhibition on CD4^+^ T cell subtypes, we performed flow cytometry on the cardiac immune cell fraction at day 7 of VM. In accordance with spleens, flow cytometry revealed that miR-155 antagonist treatment markedly reduced the activation and proliferation of CD4^+^ T lymphocytes (Fig. 7H,I). Proportions of CD4^+^CD25^+^Foxp3^+^ Tregs did not differ between treatment groups (Fig. 7J). Combined, these above data indicated that miR-155 antagonist administration could effectively rescue mice from lethal myocarditis caused by CVB3 infection.

## Discussion

In the present study, we have shown the *in vivo* role of miR-155 in CVB3-induced VM by combining a comparative study of miR-155^−/−^ KO and miR-155^+/+^ WT-infected mice. We demonstrated the up-regulation of miR-155 in the mouse model of CVB3-induced VM and that the absence of miR-155 resulted in attenuated cardiac inflammation, injury and dysfunction, and decreased mortality. The protective effect of miR-155 deficiency on VM reported here agrees with the data published by Corsten M.F.^28^. In that study, the authors showed marked amelioration of VM by a systemically delivered LNA-anti-miR-155. Our results provide further genetic evidence to support a role of endogenous miR-155 as a contributor of adverse inflammation during VM, suggesting therapeutic opportunities by targeting miR-155 in mice to limit cardiac injury and mortality in VM.

Numerous studies have indicated that the cardiac damage during VM is not mainly due to the direct cytotoxic effect of the virus on cardiomyocytes, but mostly involves the induction of immune responses^29^. Our data shows that miR-155 is an important regulator of T lymphocyte activation, and the subsequent myocardial damage, and is in line with the implication of miR-155 in other inflammatory diseases, such as EAE and rheumatoid arthritis^30^,^31^.

In the current study, silencing miR-155 reduced both cardiac damage and mortality during CVB3-induced VM, indicating that miR-155 signaling contributes to an adverse rather than beneficial immune activation in the context of VM. In the absence of miR-155, activation of spleen CD4^+^ and CD8^+^ T cells decreased, as did proliferation of spleen CD4^+^ T cells, although absolute as well as proportional numbers of CD4^+^ T cells were not changed.

Given our previous findings were in accordance with the other studies on the beneficial role of Tregs in protecting against adverse inflammation during CVB3-induced myocarditis^32^,^33^,^34^, we considered the possibility that the expansion or induction of Tregs may be involved in VM of miR-155^−/−^ mice. Surprisingly, we did not observe a difference in the proportion of Tregs in the spleens and hearts of miR-155^−/−^ and WT mice, which suggests that Tregs may not contribute to the protective effect of miR-155 silencing in VM.

Inflammatory cytokines such as TNF-α and IFN-γ, secreted mainly by pathogenic Th1 and M1 macrophages, serve as the initiator of inflammatory immune response in myocardiac disease^35^,^36^,^37^,^38^. Henke *et al.*^39^ and Nishio *et al.*^40^ showed protective effects of a Th2 cytokine, IL-10, against CVB3-induced VM. Huber and colleagues described a transgenic mice expressing TNF-α in the heart that develops severe inflammation^41^. In the present study, we demonstrated that silencing miR-155 significantly down-regulated the production of pro-inflammatory cytokines such as IFN-γ, while up-regulating the levels of anti-inflammatory cytokines such as IL-4 and IL-13 which constitute as one of the mechanism of silencing miR-155 mediated therapy of VM.

Recently, IL-17 has been shown to be pathogenic in several autoimmune disease models^42^,^43^,^44^. It was recently proposed that pathology in VM is driven by IL-17-producing CD4^+^ T cells because severe myocarditis in *T-bet* KO mice was associated with increased IL-17 expression in the heart^45^. However, we found unchanged levels of IL-17 in the hearts of miR-155^−/−^ mice, suggesting that IL-17 is not responsible for the decreased inflammation observed in the absence of miR-155.

Rather than deviating Th1/Th2/Tregs differentiation, we report here that there were no quantitative, but rather phenotypic differences in the myocardial-infiltrating macrophages between miR-155^−/−^ and WT mice following CVB3 infection. Our study reveals that macrophages of miR-155^−/−^ mice are polarized toward the M2 phenotype after CVB3 infection which could effectively suppress the development of VM. First, we observed higher myocardial IL-4 and IL-13 levels in miR-155^−/−^ mice, which are responsible for M2 polarization. Secondly, we found an increase of CD206^+^ and/or CD301^+^ macrophages that represent the alternatively-activated subset, whereas the CD206^−^CD301^−^ macrophage populations were decreased in the hearts of miR-155^−/−^ mice. Thirdly, silencing miR-155 enhanced the expression of IL-4-induced M2 markers, while potently suppressing the expression of M1 markers induced by the conditioned medium containing IFN-γ and LPS. Most importantly, our results showed that silencing miR-155 led to increased STAT6 phosphorylation, a key transcription factor involved in M2 macrophage polarization. Together, all these data suggest a central role for miR-155 in the acquisition and modulation of the M1/M2 phenotype and the regulation of miR-155 levels is important in order to exert and develop an appropriate and balanced immune response during VM.

Most of the immune mechanisms controlled by miR-155 are important in the pathogenesis of VM. In lymphocytes, miR-155 promotes Th1 responses as well as germinal center formation, while favoring proliferation and proinflammatory cytokine secretion in myeloid cells. This multitude of proinflammatory effects of miR-155 on both the innate and adaptive immune systems is associated with a variety of anti-inflammatory protein targets, including proteins suppressor of cytokine secretion 1 (SOCS1)^21^, SH2-containing inositol-5-phosphatase (SHIP1)^22^, and the transcription factors PU.1^46^ and c-MAF^47^. Further studies are required to explore the crosstalk between innate and adaptive immune cells mediated by miR-155 during VM.

In conclusion, miR-155 was up-regulated in myeloid and lymphoid inflammatory cells during VM and was a mediator of adverse cardiac immune activation. *In vivo* silencing miR-155 after CVB3 infection attenuated myocardial inflammation and necrosis, reduced mortality, and improved cardiac function. As such, this study identifies miR-155 as a novel therapeutic target for treatment of VM.

## Materials and Methods

### Mice

MicroRNA-155 knockout (miR-155^−/−^) and WT C57BL/6 male mice (Six-week-old, 16–20 g) were obtained from the Jackson Laboratory (Bar Harbor, ME, USA) or the Experimental Animal Center of Qinglongshan (Nanjing, China), and were housed in a pathogen-free mouse colonies. All animal experiments were performed according to the guidelines for the Care and Use of Laboratory Animals (Ministry of Health, China, 1998). All experimental protocols were approved by the Animal Ethical Ethical Committee of Yijishan Hospital of Wannan Medical College.

### Virus

The original stock of CVB3 (Nancy strain) was a gift from Professor Wei Hou (School of Basic Medical Sciences, Wuhan University) and was maintained by passage through HeLa cells (ATCC number: CCL-2). Virus titer was routinely determined prior to infection by a 50% tissue culture infectious dose (TCID_50_) assay of HeLa cell monolayers according to previously published procedures^41^.

### Myocarditis model and histopathology

Mice were infected by an intraperitoneal (i.p.) injection of 0.1ml of phosphate-buffered saline (PBS) containing approximately 1 × 10^5^ plaque forming units (PFU) of the virus on day 0. For *in vivo* anti–miR-155 treatment, 30 μl lipofectamine 2000 reagent was mixed with miR-155 antagonist or scrambled control (25 μg/mouse, Gene Pharma) dissolved in 170 μl PBS, and the liposome complexes were administered i.v. to mice 3 days prior to CVB3 infection. Tissue or cells were collected on day 7. Hearts were cut longitudinally and fixed in 10% phosphate-buffered formalin and embedded in paraffin. Sections (5 μm thick) were cut at various depths in the tissue section and stained with hematoxylin and eosin (H&E) to determine the level of inflammation. Sections were examined by two independent investigators blinded to the disease state of the mice.

### Real-time PCR

Total RNA was extracted from cells with TRIzol reagent (Invitrogen) and 0.4 μg RNA was used to synthesize cDNA using a first strand cDNA synthesis kit (Applied Biosystems). Real-time PCR analysis was performed using the Lightcycler 480 (Roche). The primers used are shown in Table 1. For miRNA real-time PCR, a commercial Hairpin-itTM miRNAs qPCR Quantification Kit (Gene Pharma) was used. Briefly, 2 μg RNA was used as template and then reverse-transcribed using a miR-155 specific RT-primer. The resulting cDNA was further amplified with a universal reverse primer and a specific forward primer. The PCR procedure included pre-denaturation at 95 °C for 2 min, and then 40 cycles of 94 °C for 10 seconds, 58 °C for 15 seconds and 72 °C for 20 seconds, followed by melting curve analysis. Calculations of mRNA or miRNA expression levels were performed using the comparative CT (ΔΔCT) method and normalized against *GAPDH* or *U6* snRNA levels. All reactions were run in triplicate.

### Serological index of myocarditis

Serum MB isoenzyme of creatine kinase (CK-MB) activities were measured on chemistry analyzer DXC800 (Beckman Coulter, Inc.) and serum Troponin I (cTnI) was measured on immunology analyzer DXI800 (Beckman Coulter, Inc.) by Yijishan Hospital.

### Cytokines ELISA

Cytokine levels of IFN-γ, IL-4, IL-13, IL-17, TNF-α, and IL-12 were measured in homogenized heart or cell supernatants using respective cytokine ELISA kits (R&D Systems), according to the manufacturer’s instructions.

### Plaque assay

Viral titers were determined by plaque assay as described previously^48^. Briefly, HeLa cells were seeded into six-well plates (8 × 10^5^/well) and incubated at 37 °C. Cell monolayers at ~90% confluence were washed with PBS, and then overlaid with 500μl of serial 10-fold dilutions of supernatants from heart homogenates. The cells were incubated for 1h and the supernatants removed. The cells were then overlaid with 2 ml of sterilized soft agar, incubated at 37 °C for 72 h, fixed with Carnoy’s fixative (ethanol : acetic acid = 3 : 1), for 30 min, and stained with 1% crystal violet. The plaques were counted, and the numbers of viral PFU/ml were calculated.

### *In vivo* proliferation assay

The proliferation of CD4^+^ T cells was determined by 5-bromo-2-deoxyuridine (BrdU) incorporation assay *in vivo*. BrdU (Sigma) was supplied with an injection (0.8 mg in 1 ml PBS) 1 day before termination of mice. The splenocytes were stained with conjugated anti-mouse CD4 antibody (eBioscience Inc.), fixed in IC fixation buffer (eBioscience Inc.) for 30 min and permeabilized in permeabilization buffer (eBioscience Inc.) for 20 min. Cells were then incubated at RT for 30 min in 0.15 M NaCl, 4.2 mM MgCl_2_, 10 mM HCl in the presence of 2 U DNaseI (Invitrogen), followed by staining with conjugated anti-BrdU antibody (BD Biosciences) for 30min and were finally analyzed by FACS (Beckman Coulter, Inc.).

### Echocardiographic measurements

Mice were anaesthetized by 2% isoflurane and echocardiographic examination was performed by transthoracic echocardiography with a 13 MHz transducer (GE ultrasound) on a Vingmed Vivid 7 scanner (GE ultrasound). LV diameters at end-diastole (LVEDD) and end-systole (LVESD) were measured, and fractional shortening (FS) was calculated.

### FACS analysis

Single cell suspensions were pooled from heart and spleen. Surface markers were stained with fluorochrome-conjugated mAbs diluted in 1% FBS in PBS: CD45, CD4, CD8, CD62L, F4/80, CD11b, MHCII, CD25, CD16/32, CD206, and CD301 (eBiosciences Inc.; BD Pharmingen; Biolegend). For intracellular staining, cells were fixed and permeabilized using fixation buffer and permeabilization solution or an anti-mouse Foxp3 staining kit (eBioscience Inc.). Cell fluorescence was measured using FACS (Beckman Coulter, Inc.) and data analyzed using FlowJo software (Treestar).

### Isolation and cultivation of murine BMDMs

Bone marrow cells were obtained by flushing the femurs from mice with Dulbecco’s modified Eagle’s medium (DMEM)-HEPES medium (Gibco). Cells were collected in 50 ml tubes and centrifuged for 10 min (100 × g). The supernatant was removed, and cells were suspended in DMEM (10% FCS; 20% L929 supernatant). Then, 1 × 10^6^ cells were cultured in 6-well plate at 37 °C and 5% CO2 for 7 days (M0). Macrophage polarization was obtained by removing the culture medium and culturing cells for an additional 48 h in RPMI 1640 supplemented with 5% FCS and 100 ng/ml LPS plus 20 ng/ml IFN-γ (for M1 polarization) or 20 ng/ml IL-4 (for M2 polarization).

### Western Blotting

Cells were lysed in RIPA extraction solution (15 mM Tris, pH 7.5, 120 mM NaCl, 25 mM KCl, 2 mM EGTA, 2 mM EDTA, 0.1 mM dithiothreitol, 0.5% Triton X-100, and protease inhibitor cocktail [Sigma]). Protein concentration was assessed by BCA assay. Total protein lysates (25 μg/lane) were subjected to SDS-PAGE and transferred onto an Immobilon polyvinylidene difluoride (PVDF) membrane (Millipore Corp.). Antibodies used were: anti STAT6 (Cell Signaling Technology); anti phospho-STAT6 (Cell Signaling Technology) and anti-β-actin antibody as loading control (Abcam). Western blots were quantified by Quantity One Analysis software (Bio-Rad).

### Statistical analysis

Data are shown as the mean ± SD. Statistical analysis of the data was performed with the two-tailed independent Student’s *t* test and ANOVA analysis using SPSS, version 12.0 (SPSS Inc.). The Kaplan-Meier survival curves were determined using GraphPad Prism v5.0 (GraphPad Software, Inc.). *P* < 0.05 was considered statistically significant.

## Additional Information

**How to cite this article**: Zhang, Y. *et al.* Silencing MicroRNA-155 Attenuates Cardiac Injury and Dysfunction in Viral Myocarditis via Promotion of M2 Phenotype Polarization of Macrophages. *Sci. Rep.* **6**, 22613; doi: 10.1038/srep22613 (2016).

## Figures and Tables

**Table 1:** Primer sequences used in real-time PCR are written in 5′- 3′ direction.

Genes	Primer (5′-3′)
*Nos2*	ATCTTTGCCACCAAGATGGCCTGG
	TTCCTGTGCTGTGCTACAGTTCCG
*Arg-1*	TGACTGAAGTAGACAAGCTGGGGAT
	CGACATCAAAGCTCAGGTGAATCGG
*YM-1*	ATGAAGCATTGAATGGTCTGAAAG
	TGAATATCTGACGGTTCTGAGGAG
*FIZZ1*	AGGTCAAGGAACTTCTTGCCAATCC
	AAGCACACCCAGTAGCAGTCATCCC
